# Supplementary material for: Trends in social exposure to SARS-Cov-2 in France. Evidence from the national socio-epidemiological cohort–EPICOV
Source: PLoS One. 2022 May 25;17(5):e0267725. doi: 10.1371/journal.pone.0267725 (PMC9132278; doi:10.1371/journal.pone.0267725)
Supplement: S1 Table — The national EpiCov cohort, 2020 November round. (DOCX) [file pone.0267725.s001.docx]

**Supporting S1 Table: Seroprevalence (ELISA-S > 1.1^1^) according to *département* in November 2020 among people living in mainland France ^2^. The national EpiCov cohort, 2020 November round**

| **Department** | **Name Department** | **Region** | **N** | **n** | **%** | **95% CI** |
| --- | --- | --- | --- | --- | --- | --- |
| 1 | Ain | Auvergne-Rhône-Alpes | 633 | 60 | 12.9% | [8.8-17.1] |
| 2 | Aisne | Hauts-de-France | 402 | 22 | 4.7% | [1.9-7.5] |
| 3 | Allier | Auvergne-Rhône-Alpes | 321 | 13 | 3.4% | [1.7-5.1] |
| 4 | Alpes-de-Haute-Provence | Provence-Alpes-Côte d'Azur | 286 | 13 | 3.0% | [1.3-4.7] |
| 5 | Hautes-Alpes | Provence-Alpes-Côte d'Azur | 325 | 20 | 4.7% | [1.8-7.6] |
| 6 | Alpes-Maritimes | Provence-Alpes-Côte d'Azur | 866 | 35 | 3.0% | [1.2-4.9] |
| 7 | Ardèche | Auvergne-Rhône-Alpes | 348 | 30 | 7.3% | [3.3-11.4] |
| 8 | Ardennes | Grand Est | 340 | 14 | 4.2% | [2.7-5.7] |
| 9 | Ariège | Occitanie | 276 | 7 | 1.6% | [0-3.9] |
| 10 | Aube | Grand Est | 287 | 15 | 5.8% | [3.4-8.2] |
| 11 | Aude | Occitanie | 313 | 14 | 3.8% | [1.4-6.1] |
| 12 | Aveyron | Occitanie | 305 | 8 | 3.5% | [0.6-6.3] |
| 13 | Bouches-du-Rhône | Provence-Alpes-Côte d'Azur | 1631 | 99 | 6.0% | [4.6-7.3] |
| 14 | Calvados | Normandie | 631 | 28 | 3.0% | [1.6-4.3] |
| 15 | Cantal | Auvergne-Rhône-Alpes | 293 | 9 | 2.4% | [0.8-3.9] |
| 16 | Charente | Nouvelle-Aquitaine | 343 | 7 | 2.5% | [1.1-3.8] |
| 17 | Charente-Maritime | Nouvelle-Aquitaine | 625 | 12 | 2.4% | [0.5-4.2] |
| 18 | Cher | Centre-Val de Loire | 312 | 15 | 5.2% | [2.9-7.5] |
| 19 | Corrèze | Nouvelle-Aquitaine | 297 | 13 | 5.1% | [2.4-7.8] |
| 21 | Côte-d'Or | Bourgogne-Franche-Comté | 529 | 31 | 5.8% | [3.5-8.1] |
| 22 | Côtes d'Armor | Bretagne | 539 | 16 | 1.7% | [0.8-2.7] |
| 23 | Creuse | Nouvelle-Aquitaine | 284 | 7 | 2.9% | [1.5-4.4] |
| 24 | Dordogne | Nouvelle-Aquitaine | 367 | 12 | 2.6% | [1.2-4] |
| 25 | Doubs | Bourgogne-Franche-Comté | 539 | 39 | 5.0% | [2.9-7.1] |
| 26 | Drôme | Auvergne-Rhône-Alpes | 486 | 32 | 6.9% | [4.1-9.7] |
| 27 | Eure | Normandie | 457 | 22 | 3.6% | [1.9-5.3] |
| 28 | Eure-et-Loir | Centre-Val de Loire | 357 | 18 | 5.6% | [2.8-8.5] |
| 29 | Finistère | Bretagne | 984 | 20 | 1.8% | [0.8-2.7] |
| 30 | Gard | Occitanie | 631 | 29 | 3.6% | [2.2-5] |
| 31 | Haute-Garonne | Occitanie | 1526 | 83 | 6.1% | [4.1-8.2] |
| 32 | Gers | Occitanie | 332 | 7 | 1.4% | [0.3-2.4] |
| 33 | Gironde | Nouvelle-Aquitaine | 1575 | 65 | 4.2% | [3.1-5.3] |
| 34 | Hérault | Occitanie | 1077 | 48 | 5.7% | [4.2-7.3] |
| 35 | Ille-et-Vilaine | Bretagne | 1159 | 38 | 2.7% | [1.6-3.8] |
| 36 | Indre | Centre-Val de Loire | 276 | 11 | 2.3% | [1-3.6] |
| 37 | Indre-et-Loire | Centre-Val de Loire | 604 | 18 | 2.4% | [1.4-3.4] |
| 38 | Isère | Auvergne-Rhône-Alpes | 1359 | 91 | 7.2% | [5.2-9.2] |
| 39 | Jura | Bourgogne-Franche-Comté | 324 | 19 | 7.3% | [3.9-10.7] |
| 40 | Landes | Nouvelle-Aquitaine | 377 | 15 | 4.9% | [2.9-7] |
| 41 | Loir-et-Cher | Centre-Val de Loire | 343 | 9 | 2.5% | [0-5] |
| 42 | Loire | Auvergne-Rhône-Alpes | 711 | 59 | 6.4% | [4.3-8.5] |
| 43 | Haute-Loire | Auvergne-Rhône-Alpes | 343 | 38 | 10.5% | [5.6-15.4] |
| 44 | Loire-Atlantique | Pays de la Loire | 1614 | 66 | 3.6% | [2.3-4.8] |
| 45 | Loiret | Centre-Val de Loire | 635 | 36 | 6.1% | [3.8-8.4] |
| 46 | Lot | Occitanie | 335 | 12 | 5.2% | [1-9.5] |
| 47 | Lot-et-Garonne | Nouvelle-Aquitaine | 279 | 12 | 2.4% | [0.9-3.9] |
| 48 | Lozère | Occitanie | 306 | 14 | 2.9% | [1.2-4.6] |
| 49 | Maine-et-Loire | Pays de la Loire | 788 | 35 | 3.5% | [2.1-4.8] |
| 50 | Manche | Normandie | 427 | 15 | 2.3% | [0.3-4.4] |
| 51 | Marne | Grand Est | 513 | 31 | 5.8% | [3.6-8] |
| 52 | Haute-Marne | Grand Est | 281 | 19 | 6.8% | [2.1-11.5] |
| 53 | Mayenne | Pays de la Loire | 311 | 18 | 3.9% | [1.1-6.6] |
| 54 | Meurthe-et-Moselle | Grand Est | 664 | 33 | 3.6% | [1.7-5.5] |
| 55 | Meuse | Grand Est | 315 | 20 | 4.8% | [1.6-8.1] |
| 56 | Morbihan | Bretagne | 828 | 31 | 3.5% | [1.5-5.6] |
| 57 | Moselle | Grand Est | 930 | 60 | 7.3% | [5.5-9.1] |
| 58 | Nièvre | Bourgogne-Franche-Comté | 295 | 18 | 7.6% | [3.3-12] |
| 59 | Nord | Hauts-de-France | 2207 | 185 | 7.9% | [6.2-9.5] |
| 60 | Oise | Hauts-de-France | 1678 | 107 | 6.4% | [4.7-8.1] |
| 61 | Orne | Normandie | 241 | 10 | 3.0% | [0.9-5.1] |
| 62 | Pas-de-Calais | Hauts-de-France | 1140 | 71 | 4.9% | [3.3-6.6] |
| 63 | Puy-de-Dôme | Auvergne-Rhône-Alpes | 623 | 30 | 4.9% | [2.6-7.1] |
| 64 | Pyrénées-Atlantiques | Nouvelle-Aquitaine | 615 | 30 | 4.7% | [2.4-7.1] |
| 65 | Hautes-Pyrénées | Occitanie | 317 | 10 | 4.4% | [1.1-7.7] |
| 66 | Pyrénées-Orientales | Occitanie | 365 | 17 | 5.0% | [2.6-7.5] |
| 67 | Bas-Rhin | Grand Est | 1201 | 101 | 8.0% | [5.8-10.2] |
| 68 | Haut-Rhin | Grand Est | 1607 | 181 | 9.2% | [7.2-11.2] |
| 69 | Rhône | Auvergne-Rhône-Alpes | 1963 | 168 | 10.3% | [8.1-12.5] |
| 70 | Haute-Saône | Bourgogne-Franche-Comté | 304 | 22 | 4.6% | [2.5-6.7] |
| 71 | Saône-et-Loire | Bourgogne-Franche-Comté | 467 | 29 | 6.3% | [3.7-8.8] |
| 72 | Sarthe | Pays de la Loire | 458 | 15 | 1.6% | [0.3-3] |
| 73 | Savoie | Auvergne-Rhône-Alpes | 440 | 41 | 10.1% | [5.9-14.4] |
| 74 | Haute-Savoie | Auvergne-Rhône-Alpes | 754 | 72 | 10.0% | [7.8-12.2] |
| 75 | Paris | Ile-de-France | 2339 | 249 | 11.6% | [9.7-13.4] |
| 76 | Seine-Maritime | Normandie | 1032 | 40 | 3.1% | [1.7-4.6] |
| 77 | Seine-et-Marne | Ile-de-France | 1113 | 98 | 8.0% | [5.7-10.2] |
| 78 | Yvelines | Ile-de-France | 1418 | 112 | 9.5% | [7.4-11.7] |
| 79 | Deux-Sèvres | Nouvelle-Aquitaine | 329 | 11 | 2.7% | [0.6-4.8] |
| 80 | Somme | Hauts-de-France | 449 | 33 | 9.3% | [5.6-13.1] |
| 81 | Tarn | Occitanie | 301 | 13 | 2.7% | [0.9-4.4] |
| 82 | Tarn-et-Garonne | Occitanie | 251 | 6 | 1.6% | [0.2-2.9] |
| 83 | Var | Provence-Alpes-Côte d'Azur | 748 | 28 | 3.9% | [2.3-5.4] |
| 84 | Vaucluse | Provence-Alpes-Côte d'Azur | 422 | 16 | 2.4% | [0.4-4.3] |
| 85 | Vandée | Pays de la Loire | 698 | 14 | 2.2% | [0.6-3.7] |
| 86 | Vienne | Nouvelle-Aquitaine | 414 | 9 | 1.5% | [0.2-2.7] |
| 87 | Haute-Vienne | Nouvelle-Aquitaine | 315 | 9 | 2.9% | [0.7-5.1] |
| 88 | Vosges | Grand Est | 323 | 27 | 6.7% | [4.1-9.3] |
| 89 | Yonne | Bourgogne-Franche-Comté | 282 | 11 | 2.5% | [0.8-4.2] |
| 90 | Territoire de Belfort | Bourgogne-Franche-Comté | 316 | 26 | 7.1% | [4.5-9.7] |
| 91 | Essonne | Ile-de-France | 1118 | 99 | 12.6% | [9.5-15.7] |
| 92 | Hauts-de-Seine | Ile-de-France | 1589 | 138 | 9.0% | [6.8-11.2] |
| 93 | Seine-St-Denis | Ile-de-France | 850 | 108 | 12.5% | [9.6-15.4] |
| 94 | Val-de-Marne | Ile-de-France | 1178 | 124 | 12.7% | [10.1-15.4] |
| 95 | Val-D'Oise | Ile-de-France | 836 | 93 | 12.2% | [8.4-16] |
| 2A | Corse-du-Sud | Corse | 147 | 5 | 5.0% | [1.7-8.4] |
| 2B | Haute-Corse | Corse | 142 | 4 | 4.6% | [1.4-7.8] |

**Legend of Supplementary S1 Table**

1. Home sampling by finger prick/Euroimmun ELISA-S test
2. People aged 15 years or over residing in mainland France, outside nursing homes and prisons.
3. The percentages are weighted by sampling weight (the reverse of inclusion probability), corrected for non-response probability and calibrated on the margin of the census. The prevalences are not equal to n/N.
