# Supplementary material for: Trends in social exposure to SARS-Cov-2 in France. Evidence from the national socio-epidemiological cohort–EPICOV
Source: PLoS One. 2022 May 25;17(5):e0267725. doi: 10.1371/journal.pone.0267725 (PMC9132278; doi:10.1371/journal.pone.0267725)
Supplement: S3 Table — (DOCX) [file pone.0267725.s003.docx]

**Supplementary S3 Table: Proportion of new infections between May and November 2020: proportion of positive serologies^1^ in November among people seronegative in May^2^ - The national EpiCov cohort,**

|  | **Proportion of new seropositive case** | | | | |  | **Multivariate logistic regression** | | |
| --- | --- | --- | --- | --- | --- | --- | --- | --- | --- |
|  | **N** | **n** | **%** | **95% CI^3^** | **p** |  | **ORadj** | **95% CI^3^** | **p** |
| **Overall** | **7 519** | **274** | **3.8** | **[3.1-4.7]** |  |  |  |  |  |
| Gender |  |  |  |  |  |  |  |  |  |
| Men | 3304 | 128 | 3.8 | [2.8-5.3] | 0.99 |  | Ref |  | 0.71 |
| Women | 4215 | 146 | 3.8 | [2.9-5.0] |  |  | 1.1 | [0.7-1.6] |  |
| Age (years) |  |  |  |  |  |  |  |  |  |
| 15-17 | 179 | 9 | 4.3 | [1.2-14.2] | 0.001 |  | 5.0 | [0.8-16.0] | <0.001 |
| 18-24 | 532 | 40 | 9.2 | [5.2-15.6] |  |  | 4.2 | [1.2-15.0] |  |
| 25-34 | 841 | 46 | 4.6 | [3.0-7.1] |  |  | 1.6 | [0.4-6.3] |  |
| 35-44 | 1180 | 43 | 2.8 | [1.7-4.4] |  |  | 0.9 | [0.2-3.7] |  |
| 45-54 | 1498 | 55 | 4.8 | [3.1-7.4] |  |  | 2.5 | [0.6-9.4] |  |
| 55-64 | 1519 | 50 | 3.3 | [2.2-5.1] |  |  | 1.8 | [0.5-6.7] |  |
| 65-74 | 1242 | 22 | 1.4 | [0.8-2.8] |  |  | 0.8 | [0.2-3.0] |  |
| 75+ | 528 | 9 | 1.6 | [0.5-4.7] |  |  | Ref |  |  |
| Migration status ^4^ |  |  |  |  |  |  |  |  |  |
| No (majority population) | 6268 | 213 | 3.7 | [2.8-4.7] | <0.001 |  | Ref |  | 0.007 |
| First- generation from Europe | 224 | 2 | 0.2 | [0.04-1.5] |  |  | 0.1 | [0.01-0.6] |  |
| Second- generation Europe | 413 | 14 | 1.9 | [0.7-4.9] |  |  | 0.7 | [0.2-1.9] |  |
| First-generation from outside Europe | 272 | 21 | 5.4 | [2.9-10.0] |  |  | 1.7 | [0.8-3.9] |  |
| Second- generation outside Europe | 263 | 21 | 9.9 | [5.5-17.1] |  |  | 2.8 | [1.2-6.1] |  |
| Occupational status ^5^ |  |  |  |  |  |  |  |  |  |
| Healthcare profession | 355 | 28 | 7.1 | [4.0-12.0] | 0.17 |  | 2.3 | [1.2-4.7] | 0.09 |
| Other essential profession | 683 | 26 | 4.6 | [2.6-7.9] |  |  | 1.2 | [0.6-2.6] |  |
| Non-essential profession | 3052 | 120 | 4.2 | [2.9-6.1] |  |  | Ref |  |  |
| No occupation | 3428 | 100 | 3.2 | [2.3-4.4] |  |  | 1.0 | [0.5-2.0] |  |
| Educational level |  |  |  |  |  |  |  |  |  |
| < High school diploma | 1045 | 28 | 1.9 | [1.0-3.5] | 0.03 |  | Ref |  | 0.05 |
| High school diploma | 2366 | 78 | 4.0 | [2.8-5.6] |  |  | 2.5 | [1.1-5.7] |  |
| Secondary first-degree diploma | 1518 | 58 | 5.4 | [3.2-8.9] |  |  | 3.2 | [1.4-7.2] |  |
| ≥ Bachelor’s degree | 2590 | 110 | 4.7 | [3.4-6.3] |  |  | 2.5 | [1.1-6.0] |  |
| Family income per capita (deciles) |  |  |  |  |  |  |  |  |  |
| D01(lowest) | 395 | 16 | 3.1 | [1.5-6.2] | 0.88 |  | 0.5 | [0.2-1.3] | 0.79 |
| D02-D03 | 776 | 29 | 4.6 | [2.5-8.5] |  |  | 1.0 | [0.5-2.0] |  |
| D04-D05 | 985 | 35 | 3.7 | [2.3-5.9] |  |  | 1.0 | [0.5-2.0] |  |
| D06-D07 | 1522 | 46 | 3.6 | [2.4-5.5] |  |  | Ref |  |  |
| D08-D09 | 2258 | 80 | 3.4 | [2.4-4.9] |  |  | 1.0 | [0.5-1.7] |  |
| D10 | 1428 | 60 | 4.0 | [2.6-6.3] |  |  | 1.0 | [0.5-2.0] |  |
| Tobacco use |  |  |  |  |  |  |  |  |  |
| Daily smoker | 1159 | 28 | 2.9 | [1.6-5.1] | 0.34 |  | Ref |  | 0.57 |
| Occasional smoker | 349 | 14 | 3.9 | [1.8-8.3] |  |  | 1.1 | [0.4-3.3] |  |
| Ex-smoker | 1937 | 61 | 3.2 | [2.2-4.6] |  |  | 1.5 | [0.7-3.0] |  |
| Non-smoker | 4067 | 171 | 4.4 | [3.5-5.9] |  |  | 1.6 | [0.8-3.0] |  |
| Population density in municipality |  |  |  |  |  |  |  |  |  |
| Low | 2340 | 69 | 2.2 | [1.6-3.2] | 0.020 |  | Ref |  | 0.048 |
| Medium | 2248 | 82 | 4.7 | [3.0-7.4] |  |  | 1.9 | [1.1-3.3] |  |
| High | 2931 | 123 | 4.6 | [3.5-6.0] |  |  | 1.6 | [0.9-2.9] |  |
| Socially deprived neighbourhood |  |  |  |  |  |  |  |  |  |
| No | 7246 | 257 | 3.4 | [2.8-4.2] | 0.005 |  | Ref |  | 0.028 |
| Yes | 273 | 17 | 10.6 | [4.8-22.0] |  |  | 2.7 | [1.1-6.7] |  |
| Number of people in household |  |  |  |  |  |  |  |  |  |
| 1 | 1287 | 39 | 4.1 | [2.6-6.4] | 0.63 |  | Ref |  | 0.75 |
| 2 | 3004 | 90 | 3.2 | [2.1-5.0] |  |  | 0.7 | [0.4-1.4] |  |
| 3 | 1260 | 52 | 3.5 | [2.2-5.6] |  |  | 0.6 | [0.3-1.3] |  |
| 4 | 1357 | 69 | 4.4 | [3.1-6.3] |  |  | 0.7 | [0.4-1.4] |  |
| 5 or more | 606 | 24 | 5.2 | [2.8-9.5] |  |  | 0.8 | [0.4-2.0] |  |

**Legend of S3 Table**

1. Home sampling by finger prick/Euroimmun ELISA-S test
2. People aged 15 years or over residing in mainland France, outside nursing homes and prisons.
3. The sampling design is taken into account for the estimation of prevalence, confidence intervals (logit transformation), crude and adjusted odds ratios, confidence intervals and statistical tests, with the SAS procsurvey procedure. The percentages are weighted by sampling weight (the inverse of inclusion probability), corrected for non-response weigts and calibrated on the margin of the census. The prevalences are not equal to n/N. .
4. Migratory status: Majority population = persons born in France who are neither first nor second-generation immigrants / First-generation immigrants: born non-French outside France and living permanently in France (including those who subsequently acquired French nationality) / Second-generation immigrants: born and living in France, with at least one parent being a first-generation immigrant
5. Self-reported in round 1: a) Healthcare professions Included medical and paramedical professionals, Firefighters, pharmacists and ambulance drivers (but not including hospital cleaners, for example).; b) Other essential profession included: Home helps or housekeepers, food shop workers, delivery drivers, public transportation drivers, cab drivers, bank customer service or reception staff, petrol station employees, police officers, postal workers, cleaning staff, security guards, construction workers, truck drivers, farmers and social workers), also self-reported.
