# Supplementary material for: Trends in social exposure to SARS-Cov-2 in France. Evidence from the national socio-epidemiological cohort–EPICOV
Source: PLoS One. 2022 May 25;17(5):e0267725. doi: 10.1371/journal.pone.0267725 (PMC9132278; doi:10.1371/journal.pone.0267725)
Supplement: Attachment — Submitted filename: 0-Rebutal_letter_24March2022_plosone.docx [file pone.0267725.s004.docx]

**RESPONSES TO EDITOR and REVIEWER**

**New editor comments (march 2022)**

**A)** Can you make a de-identified, anonymized, or aggregated data set publicly available in a repository or, if that is not possible, in your manuscript’s Supporting Information files? For a list of recommended repositories, some of which are able to hold sensitive data, see here: <https://journals.plos.org/plosone/s/recommended-repositories>

**Response:** All aggregated data set concerned the submitted paper is available online and on supplementary files already submitted, and not only for the first round as written by error. This was similar than in a previous study from EpiCov, that we published on Plos One : DOI: [10.1136/bmjopen-2021-052888](https://doi.org/10.1136/bmjopen-2021-052888). Changes was made in the statement form

The second information on data availability did not concern access to the data used in the submitted paper, as all aggregated data are available in the submission. It concerned access to the EpiCov study for researchers. Researchers who meet the criteria, after regulatory approval, have access on a secured platform to analyse the data, with only extraction of aggregated data.

The French law forbids us to provide free access to individual EpiCov data, under the protection of health data regulation set by the French National Commission on Informatics and Liberty.

We then clarified the availability statement : **“All anonymous aggregated data concerning the results presented in this paper are available online and on supporting information files.** **The EpiCov study is available for research purpose after submission to approval of French Ethics and Regulatory Committee procedure (Comité du Secret Statistique, CESREES and CNIL). Access procedure is available on CASD (**[**https://www.casd.eu/**](https://www.casd.eu/)**). Additional information can be addressed to the corresponding author.”**

b) Are there any institutional representatives who did not collaborate in the study and are not listed as authors on the manuscript who would be able to hold the data and respond to external requests for data access? If so, please provide their contact information (i.e., email address). Please also provide details on how you will ensure persistent or long-term data storage and availability. For instance, data might be stored in two independent locations.

**Response**: The EpiCov study is under the joint responsibility of the National Institute of Health and Research (INSERM) depending on the Ministry for Research, as Promotor, and the Direction of Research, Studies and Statistical Evaluation depending (DREES) depending on Ministry of Health, as indicated in sponsor. A representative of the INSERM promotor can be joined about the EpiCov study: Karim Ammour, Inserm- Pôle de Recherche Clinique/Clinical Research Department, BIOPARK 8 rue Croix de Jarry;  75013  Paris Tel. +33(0)144236067 [karim.ammour@inserm.fr](mailto:karim.ammour@inserm.fr)

Data are stored on two independent secured plaforms depending on each of the two responsible Institution, and long -term storage is planned for 15 years, according to the French Regulation, as declared and approved by the regulatory authorities mentioned in the statement form (Ethics approval)

**Editor comments**

**Q2 and 3 Financial disclosure** :

Role of the funders:

Response: The funders had no role in study design, data collection and analysis, decision to publish, or preparation of the manuscript

Place in the manuscript:

Response: the contains is strictly similar to those provided in the online Funding Statement and we removed it from the manuscript, and we will add the above sentence about the role of the funders

Added in the cover letter

This research was supported by research grants from Inserm (*Institut National de la Santé et de la Recherche Médicale*) and the French Ministry for Research, by Drees-*Direction de la Recherche, des Etudes, de l’Evaluation et des Statistiques*, and the French Ministry for Health, and by the Région Ile de France.

Dr. Bajos has  received funding from the European Research Council (ERC) under the European Union’s Horizon 2020 research and innovation programme (grant agreement No. [856478])

This project has also received funding from the European Union’s Horizon 2020 research and innovation programme under grant agreement No 101016167, ORCHESTRA (Connecting European Cohorts to Increase Common and Effective Response to SARS-CoV-2 Pandemic).

**The funders had no role in study design, data collection and analysis, decision to publish, or preparation of the manuscript.**

**Q4 Title** : Please amend either the title on the online submission form (via Edit Submission) or the title in the manuscript so that they are identical.

Response: keep title in the manuscript

**Trends in social exposure to SARS-Cov-2 in France. Evidence from the national socio-epidemiological cohort – EPICOV**

**Q5 Group epicov :** One of the noted authors is a group or consortium Epicov Team. In addition to naming the author group, please list the individual authors and affiliations within this group in the acknowledgments section of your manuscript. Please also indicate clearly a lead author for this group along with a contact email address

Response: Done

**Q6 “data not shown” in the manuscript :**

Response: Such data are not necessary for the current paper and they are developed in a submitted paper currently in revision elsewhere. We then suppressed the sentence which referred to it as it is not necessary. Our discussion and conclusion are entirely supported by data included in the paper (line 336 to 341).

**We suppressed the unnecessary sentence in the discussion “Populations of non-European first and second-generation immigrants were as compliant with barrier measures as others in March and November (data not shown)”.**

**Q7 and 8 Please upload a new copy of Figure 2 as the detail is not clear. We note that Figure 2 in your submission contain [map/satellite] images which may be copyrighted.**

- Response: It was too difficult to obtain total copyright. This figure is not needed as the Table 1 and the supplementary table (as referred) bring geographical seroprevalence.
- **We suppressed the figure 2**

**Additional Editor Comments:**

**Minor comments**

**1. please disucss the paper below.**
Lee SW, Yuh WT, Yang JM, Cho YS, Yoo IK, Koh HY, Marshall D, Oh D, Ha EK, Han MY, Yon DK. Nationwide Results of COVID-19 Contact Tracing in South Korea: Individual Participant Data From an Epidemiological Survey. JMIR Med Inform. 2020 Aug 25;8(8):e20992. doi: 10.2196/20992. PMID: 32784189; PMCID: PMC7470235.

Response : please, can you explain what is expected as discussion?

**2.Title**
Trends in social exposure to SARS-Cov-2 in France. Evidence from the national socioepidemiological cohort – EPICOV Evidence from the national socio-epidemiological cohort – EPICOV->
Trends in social exposure to SARS-Cov-2: Results from the French Nationwide Cohort

Response: we retain “Trends in social exposure to SARS-Cov-2 in France. Evidence from the national socio-epidemiological cohort – EPICOV”

**Reviewer #1**

I thank the reviewer for the comments and enclose here our responses to proposals or questions.

**Com1 :** Keywords: I suggest to the authors to exclude some keywords as “random sample” and include keywords that remind the country the study was conducted.
Response: We think that the specificity of such design is very important to mention in keywords and propose to replace random sample by “probability sample design”. The country is mentioned in the title.

**Keyword : random sample replaced by “probability sampling design”**

**Com2 :** “African, Asian and other ethnic minorities were disproportionately affected by SARS-CoV-2 in Europe and North America during the first epidemic wave.” Please include “Latin-American individuals”

Response: Done

**Added L62: African, Asian, Latin-American and other ethnic minorities were disproportionately affected by SARS-CoV-2 in Europe and North America during the first epidemic wave**

**Com3 : “**France has been severely affected by COVID-19. The first wave peaked two weeks after the first lockdown initiated on 17th March” Please describe here the year. Also please describe in the text if it was a national lockdown or regional lockdown. The second wave started slowly at the end of August,” Same observation as described above regarding the year.

Response: Done

**The first national lockdown initiated on 17th March 2020 (line 68)**

**A second national lockdown was instated from 30 October to 15 December 2020 (line 76)**

**Com6 :** “ but leaving more opportunities to get together from the summer.” Please clarify this statement

Response: I agree that it is not very clear. the paragraph was clarified

**Change line 77-82 : Unlike the first lockdown which caused widespread suspension of both social and professional life, the second was less restrictive, with no school closure and extended list of shops authorized to remain open. Between the first and second lockdown, teleworking was encouraged, measures maintaining barriers to extra-professional social life remained, especially face covering and maximum numbers admitted to access attractions, coffees and restaurant, but which let more opportunities to get together, especially during the summer**.

**Com7 :** Please describe what is “FIDELI administrative sampling framework” ?

Response: done

102 to 105 : **FIDELI is the national database on housing and individuals issued from tax files, containing demographic information on people and household structure and income, and additional contextual data about the living place of people.**

**Com7 :** “Residents in nursing homes for elderly persons were excluded.” Please describe why.

Response: done

- Added in lines 107 to 108 : **Residents in nursing homes for elderly persons were excluded, as it was not feasible to obtain help from caregivers to facilitate telephone or web contact with them during the first lockdown.**

**Com9:** In the Exposure section were evaluated medical conditions of the participants? It is not clear for me.

Response: Self reported symptoms and comorbidities were collected in the questionnaire. For this analysis, we did not include data on symptoms. Adjustment for some comorbidities to study the relation of seropositivity with migration status was performed and presented in supplemental material.

We added in line 136 : **Individual characteristics included …, body max index and comorbidities…..**

**Com9:** Results section is well-described. Tables are adequate too. I would suggest to exclude table 4 and describe its information in the text, due to a lot of tables in the study.

Response: done

- **The Table 4 was removed and added to supplemental data S3**

**Com11:** Discussion: The discussion section is well argued. I miss other data in the literature on serological surveys in France and in countries that have adopted the lockdown, such as England and Spain.

Response: done

Added in line 424 et 425 (**with two added references) : The two other national serological studies based on random general population samples was conducted in Spain (ref Pollan) and England (ref Ward) and reported prevalence of same magnitude than in France**.

**Reviewer #2**

I thank the reviewer for the comments and enclose here our responses to proposals or questions.

**Com1 :** The authors talk about univariate and multivariate analyses, but a much more extensive explanation should be provided. Which statistical tests have been applied, how, and why? Do the data fulfill all requirements to apply these tests? What about the statistical potency? I guess it is high due to the very high sample number. Please, show all details. Please, present the data in an APA format or similar, associating the statistical value to the p-values that appear in tables. I guess the t-test has been applied because the confidence intervals are given, but statistical analysis is of utmost importance here and should be explained very rigorously. I guess that it has been properly performed, but an idea only exist as far as it is written, as Jacques Monod highlighted. The a priori chosen level of statistical significance should be indicated. The same is applicable to correlation and logistic regression analysis and inference.

Response:

I agree with the importance to add more details on the statistical methods used in this paper (that I reduce because of the limits of words).

The methodology is adapted to complex sample design, as standard procedures based upon classical SRS (simple random sample) and IID (Independent and identically distributed random variables) are generally not appropriate in such design. There is a large amount of methodological literature and I added two major classical references on the principle of the méthods in that domain, with large discussion on design-based or model-based approach:

- Skinner CJ, Holt D, Smith TMF. Analysis of complex surveys [Internet]. John Wiley & Sons; 1989 [cited 2022 Feb 12]. 328 p. Available from: https://eprints.soton.ac.uk/34690/
- Rao JN, Scott AJ. On chi-squared tests for multiway contingency tables with cell proportions estimated from survey data. Ann Stat. 1984;46–60.

We used here the design-based statistical methods, classically used in many population-based study, using procedure developed and validated in Stata (svy procedures), SAS (proc survey), or R (package survey)

I am not used to add the value of the statistics value with the p-value, and which is rarely presented in epidemiologic papers. Statistical value is not interpretable here as it does not correspond to classical tests. Moreover tests and confidence intervals cannot be calculated from frequency presented in the tables as weighting is applied for point estimate, and design is taken into account for variance estimation.

I rewrited the methodological paragraph with reference to the two papers, and hope it will be sufficient.

Paragraph rewritten (line 171-177): **The unequal probabilities sampling design, and final calibrated weights were taken into account, with the specific design-based “proc survey” procedures of SAS and “svy” procedures of STATA. Prevalences were estimated, using weighted percentages, and logit transformed confidence limits were used to remain within the interval [0,1]. The design-based Pearson chi-squared test statistic developed by Rao was used for multiway contingency tables (12). Crude and adjusted odds ratios were estimated with logistic regression models based on design-based methods (11).** **The significance threshold was 0.05.**

**Com2 :** What about the statistical potency? I guess it is high due to the very high sample number.

Response: Sample size was initially calculated so as to ensure sufficient precision for the seroprevalence estimate, the goal being to obtain a 95% confidence interval of 2 points for a prevalence of 5% in administrative subdivisions of 600,000 inhabitants (*department* or *metropolitan area*). Moreover individuals living in a household below the poverty line were overrepresented to have sufficient powerful to study relation of exposure with social disadvantage (as indicated in line 107-108)

**Com2 :** As the authors point out in the "Limitations" section, circulating antibody titles may vary and decay over time, and even they disappear in certain cases. However, memory B cell analysis is not feasible in this context. In lines 385 through 387, the authors highlight consistency between factors associated to incidence and prevalence. However, this does not solve the limitation, and this should be also pointed out. In this sentence, "of new infections" should be removed, because this is included in the "incidence" concept.

Response: We agree that the decline of antibodies should be a limitation to study trends in prevalence. However, our objective was to study whether there were changes in population exposed to Covid between first and second epidemic waves. As our conclusions are very similar when analyzing new infection between May and November and when comparing factors associated with prevalences at each period, we can conclude than decline in antibodies was not a source of bias for our main results.

**Paragraph changed in 412-16 :** **However, such decline seems not to be a source of bias to study changes is population exposed to covid between the two epidemic waves: our results were similar when analysing factors associated with new Covid infections between May and November in the subsample tested in both rounds, and changes in factors associated with seroprevalence between these two periods.**
